# Supplementary material for: Novel Thymoquinone Nanoparticles Using Poly(ester amide) Based on L-Arginine-Targeting Pulmonary Drug Delivery
Source: Polymers (Basel). 2022 Mar 8;14(6):1082. doi: 10.3390/polym14061082 (PMC8956027; doi:10.3390/polym14061082)
Supplement: Supplementary file 1 [file polymers-14-01082-s001.zip › polymers-1578819-supplementary-done.pdf]

polymers

MDPI

Supplementary Materials

Novel Thymoquinone Nanoparticles Using Poly(Ester Amide) Based on L-Arginine-Targeting Pulmonary Drug Delivery

Eman Zmaily Dahmash [1,*], Dalia Khalil Ali [2], Hamad S. Alyami [3,*], Hussien AbdulKarim [1], Mohammad H. Alyami [3] and Alhassan H. Aodah [4]

[1] Department of Applied Pharmaceutical Sciences and Clinical Pharmacy, Faculty of Pharmacy, Isra University, Amman, 11622 Jordan; eman.zmaily@iu.edu.jo; hussein.abdalkarem@yahoo.com
[2] Department of Physiotherapy Department, Faculty of Allied Medical Sciences, Isra University, Amman, 11622 Jordan; dalia.ali@iu.edu.jo
[3] Department of Pharmaceutics, Faculty of Pharmacy, Najran University, Najran, 55461 Saudi Arabia; mhalmansour@nu.edu.sa
[4] National Center of Biotechnology, Life Science & Environment Research Institute, King Abdulaziz City for Science and Technology, Riyadh, 11442 Saudi Arabia; aaodah@kacst.edu.sa
* Correspondence: eman.zmaily@iu.edu.jo (E.Z.D.) ORCID https://orcid.org/0000-0002-9815-3720; hsalmukalas@nu.edu.sa (H.S.A.) ORCID https://orcid.org/0000-0003-3513-4976.; Tel.: +962-797439871 (E.Z.D.); +966-6175417964 (H.S.A.)

Section S1

S1.1 Release Kinetic Modelling

Figures S1-S5 demonstrated the linear regression of the five release kinetics mathematical models.

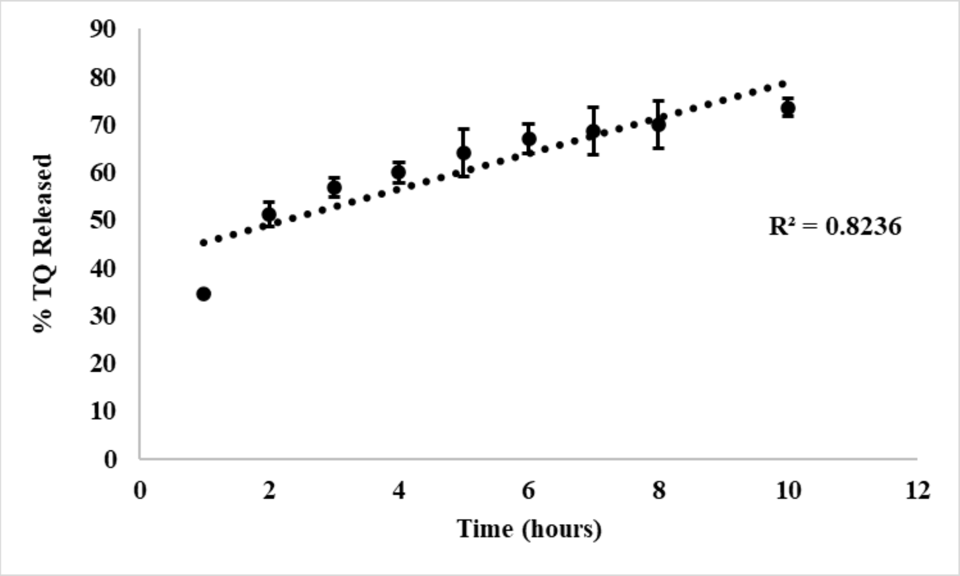

Figure S1. Linear regression of Zero Order Kinetics mathematical model of TQ from TQ-loaded poly(ester amide) based on L-arginine nanoparticles (F3) with the related R^2 value.

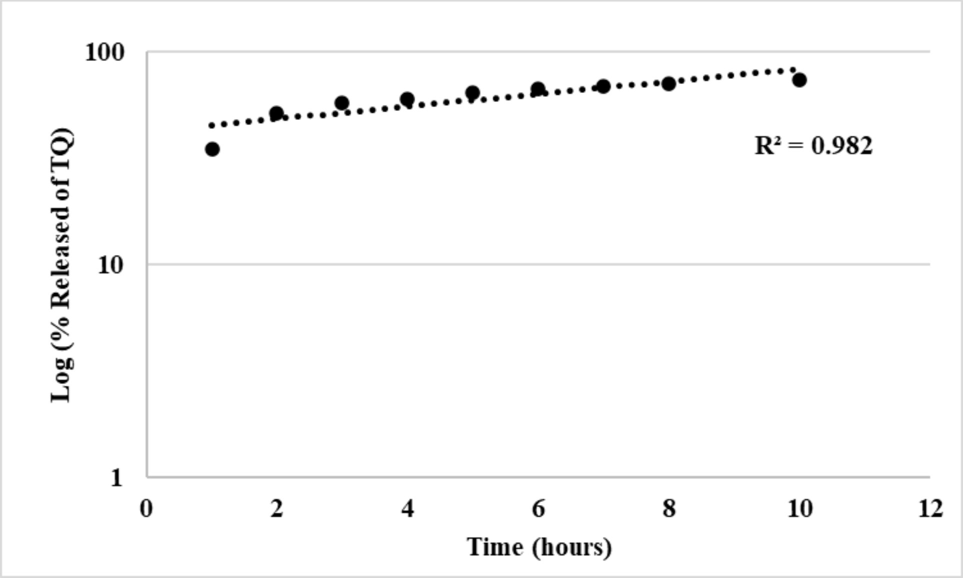

Figure S2. Linear regression of First Order Release Kinetics mathematical model of TQ from TQ-loaded poly(ester amide) based on L-arginine nanoparticles (F3) with the related R^2 value.

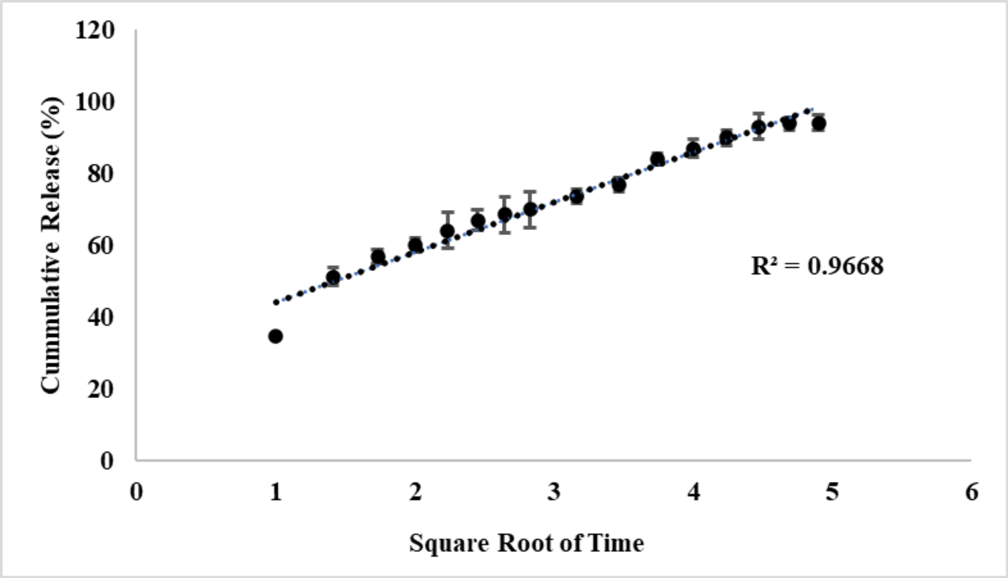

Figure S3. Linear regression of Higuchi Release Kinetics mathematical model of TQ from TQ-loaded poly(ester amide) based on L-arginine nanoparticles (F3) with the related R^2 value.

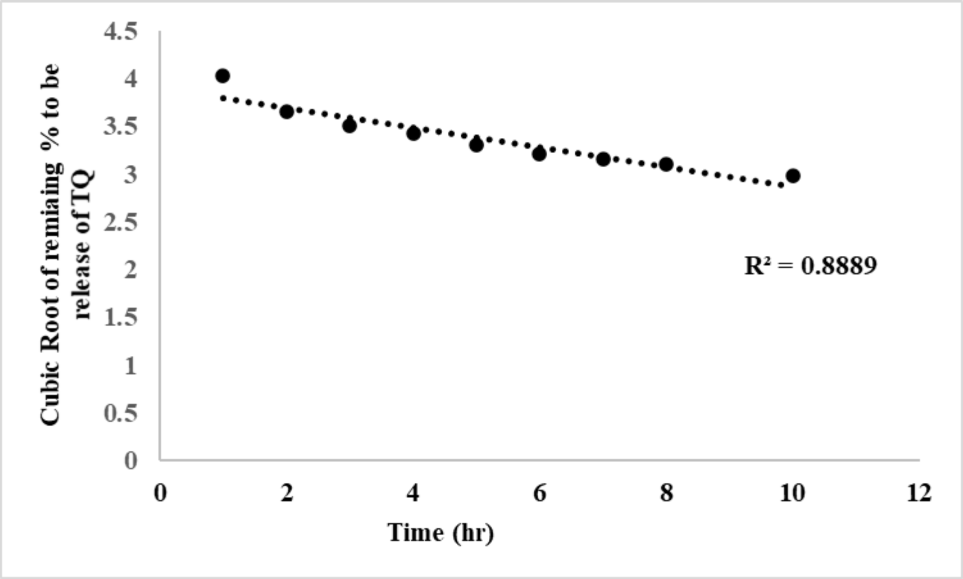

Figure S4. Linear regression of Hixon Crowell Release Kinetics mathematical model of TQ from TQ-loaded poly(ester amide) based on L-arginine nanoparticles (F3) with the related R^2 value.

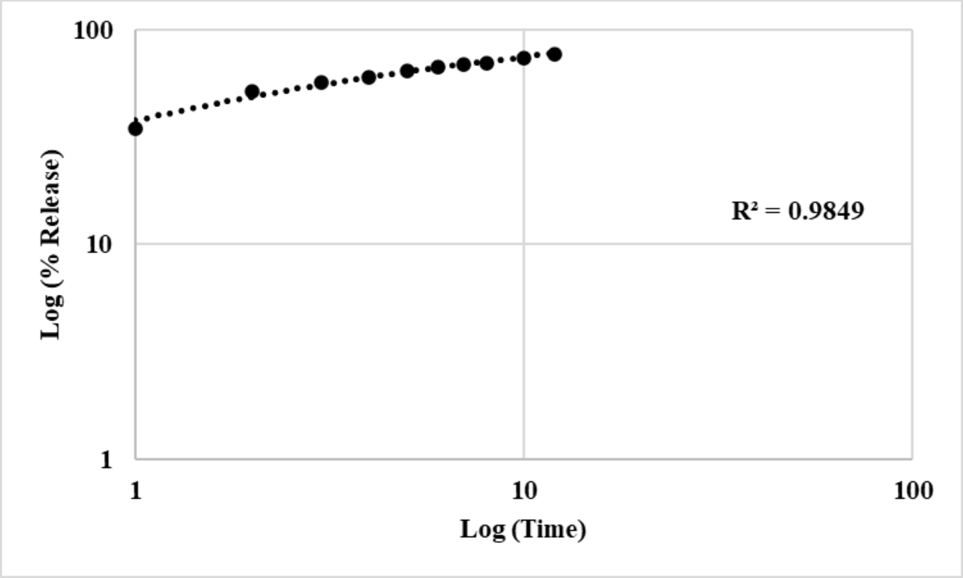

Figure S5. Linear regression of Korsmeyer -Peppas Release Kinetics mathematical model of TQ from TQ-loaded poly(ester amide) based on L-arginine nanoparticles (F3) with the related R^2 value.
